# Pharmacomechanical Thrombectomy and Catheter-Directed Thrombolysis, with or without Iliac Vein Stenting, in the Treatment of Acute Iliofemoral Deep Vein Thrombosis

**DOI:** 10.3390/jcdd11070214

**Published:** 2024-07-09

**Authors:** Evren Ozcinar, Nur Dikmen, Ahmet Kayan, Melisa Kandemir, Mehmet Cahit Saricaoglu

**Affiliations:** 1 Department of Cardiovascular Surgery, Heart Center, Cebeci Hospitals, Ankara University School of Medicine, Ankara 06230, Turkey; evrenozcinar@gmail.com (E.O.); melisakandemir1999@gmail.com (M.K.); cahitsarica@gmail.com (M.C.S.); 2 Department of Cardiovascular Surgery, Kirikkale High Specialization Hospital, Kirikkale 71300, Turkey; dr.ahmet.kayan@gmail.com

**Keywords:** acute deep vein thrombosis, pharmacomechanical thrombectomy, catheter-directed thrombolytic, venous stent, endovascular procedures

## Abstract

Background: This study aims to evaluate and compare the outcomes and clinical efficacy of pharmacomechanical thrombectomy (PMCT) plus catheter-directed thrombolysis (CDT) and PMCT combined with CDT and venous stenting in managing acute iliofemoral deep vein thrombosis (DVT), while also assessing the long-term safety and efficacy of these interventions. Methods: A retrospective case–control study spanning 3 years involved 112 patients presenting with acute symptomatic iliofemoral deep vein thrombosis (DVT), each with a symptom duration of less than 14 days. Patients were consecutively categorized into two groups based on individual clinical indications: PMCT + CDT vs. PMCT + CDT + venous stent. Statistical analyses were conducted to compare clinical features and outcomes between the two groups. Additionally, patients were followed up for 24 months post-treatment, during which quality of life (QoL) and severity of post-thrombotic syndrome (PTS) were analyzed. Results: In this retrospective study, we analyzed a total of 112 consecutive patients, with 63 patients undergoing PMCT + CDT and 49 patients undergoing PMCT + CDT + venous stent. Between the two groups, regarding primary outcomes at 6 months, there was no difference in the observed cumulative patency rates, standing at 82.5% for PMCT + CDT and 81.6% for PMCT + CDT + stent. Survival analyses for primary, primary-assisted, and secondary patency yielded comparable results for PMCT + CDT, with *p*-values of 0.74, 0.58, and 0.72, respectively. The two-year patency rate was high in both groups (85.7% for PMCT + CDT vs. 83.7% for PMCT + CDT + stent). Additionally, during the follow-up period, there were no statistically significant differences observed in the incidence of PTS or the average Villalta score between the two groups. At 24 months post-intervention, the incidence of post-thrombotic syndrome (PTS) was 11.1% in the PMCT + CDT group and 22% in the PMCT + CDT + stent group (*p* = 0.381). Both treatment arms of the study groups experienced bleeding complications during the thrombolysis therapy; in the PMCT + CDT group, there were three cases of gastrointestinal bleeding, compared to two cases in the PMCT + CDT + stent group (*p* = 0.900). Additionally, there was one intracranial hemorrhage in the PMCT + CDT group and two in the PMCT + CDT + stent group. Conclusions: Pharmacomechanical thrombectomy (PMCT) combined with catheter-directed thrombolysis (CDT) therapy has shown significant efficacy in alleviating leg symptoms and reducing the occurrence of post-thrombotic syndrome (PTS), including the incidence of moderate-to-severe PTS. On the other hand, the utilization of PMCT + CDT + stent therapy, tailored to individual patients’ clinical and venous conditions, may enhance long-term venous patency and lead to superior outcomes, including improved quality of life parameters.

## 1. Introduction

Deep vein thrombosis (DVT) stands as a significant and severe health concern, impacting approximately 1 individual per 1000 people annually. It is regarded as the third most prevalent cause of cardiovascular morbidity [1,2,3]. Patients diagnosed with acute deep vein thrombosis (DVT) affecting the iliofemoral vein segment experience the poorest outcomes in terms of post-thrombotic syndrome (PTS) and quality of life (QoL) [4,5]. Additionally, patients diagnosed with deep vein thrombosis (DVT) are predominantly managed with anticoagulation and compression therapy [6]. Deep vein thrombosis (DVT) is often underestimated as an illness, yet it carries significant morbidity, often culminating in post-thrombotic syndrome (PTS). Remarkably, around 50% of DVT patients eventually develop PTS. This syndrome is recognized as a chronic and progressive condition, potentially resulting in venous ulceration and a notable decline in the quality of life associated with the affected disease [7,8].

Over the past decade, numerous studies and several large multi-center trials have increasingly endorsed the efficacy of catheter-directed thrombolysis (CDT) and pharmacomechanical thrombectomy (PMCT) in managing acute iliofemoral deep vein thrombosis (DVT). These interventions aim to promptly remove thrombosis during the early stages of venous disease [9]. Despite the promising outcomes of percutaneous thrombus treatment modalities such as pharmacomechanical thrombectomy (PMCT) and catheter-directed thrombolysis (CDT) for acute iliofemoral deep vein thrombosis (DVT), patients diagnosed with chronic residual iliofemoral lesions resulting from acute DVT are often undertreated or overlooked. While published data suggest the efficacy of CDT and PMCT in treating acute iliofemoral DVT, the role of venous stenting in this context remains unclear. Furthermore, conflicting results have cast uncertainty on the current role of venous stenting in addition to combined CDT and PMCT therapies. Although venous stenting may complement catheter-directed therapies in real-world acute DVT cases, the lack of comparative data prevents conclusions regarding the relative benefits of these different modalities [10,11,12,13,14,15].

This retrospective cohort study conducted at a single center sought to evaluate the outcomes of patients treated with PMCT (AngioJet thrombectomy using the AngioJet Zelante DVT catheter; Boston Scientific, Marlborough, MA, USA) in addition to CDT (Cragg–McNamara catheter of appropriate length (Medtronic, Watford, UK)) vs. patients treated with PMCT, CDT, and adjunctive venous stent placement using the Abre self-expanding venous stent (Minneapolis, MN, USA).

## 2. Material and Methods

### 2.1. Patient Selection and Features

Between 2018 and 2022, a total of 112 (CDT + PMCT: *n* = 63; and PMCT + CDT + stent: *n* = 49) patients with symptomatic acute iliofemoral DVT < 14 days of symptom duration were enrolled. The research ethics board approved this study. This study was conducted in accordance with the principles of the Declaration of Helsinki. Patients with a history of adverse reactions to the treatment of anticoagulation or thrombolysis were excluded. Exclusion criteria also covered active bleeding or bleeding from unknown or undiagnosed origin, intracranial hemorrhage, anemia, lower creatinine clearance (<30 mL/min), pregnancy, thrombocytopenia, and patients <18 years old (11). All patients provided informed consent before treatment initiation. We retrospectively reviewed patient demographic data, symptom characteristics and duration, stent placement specifics, anticoagulation treatment approaches, post-intervention vessel patency duration, and postoperative Villalta scores.

### 2.2. Procedural Technique

Upon admission, duplex ultrasonography and computed tomography were utilized to assess the extent of venous thrombosis and establish the diagnosis. All treated patients were symptomatic and received anticoagulant therapy.

Treatment selection was based on patients’ symptoms and underlying residual iliofemoral venous vessel disease. For both treatment modalities, venous access was established via the popliteal vein using the ultrasound-guided puncture technique. Guidewires were then positioned into the inferior vena cava prior to treatment initiation. Pharmacomechanical thrombectomy (PMCT) was performed using the Angiojet system according to the manufacturer’s instructions. PMCT technique involved power pulse followed by aspiration mode using AngioJet, followed by catheter-directed thrombolysis (CDT) administered over a duration of 12–24 h.

For the PMCT + CDT group, successful lysis completion was defined as achieving >90% clearance of the clot, as determined by venographic criteria. In the PMCT + CDT + stent group, the presence of residual stenotic venous disease, extrinsic compression, and the need for stenting were evaluated through venographic examination, as IVUS catheters are not available in our country. If an obstructive lesion or residual venous disease was identified, it was addressed through balloon venoplasty and subsequent stent placement, following the procedural steps of PMCT + CDT interventions.

All patients in both groups underwent continuous monitoring of anticoagulation levels throughout the procedure. Intravenous heparin was administered to maintain an activated clotting time (ACT) exceeding 250 s. Manual compression was initiated alongside lytic therapy and continued until discharge. After completing the lysis, intervention introducers were removed, and compression stockings were applied. Therapeutic low-molecular-weight heparin (LMWH) was promptly initiated within one hour post-procedure. Patients were prescribed one month of therapeutic LMWH, transitioning to oral anticoagulants following a two-week surveillance ultrasonography. In the majority of cases, patients were subsequently managed with direct oral anticoagulants (DOACs).

### 2.3. Measurement of Outcomes

The primary outcomes assessed included venous patency at 3, 6, 12, 18, and 24 months, the incidence of post-thrombotic syndrome (PTS), and the presence of valvular dysfunction identified via ultrasound. The 3-month interval follow-up emerged as the most accurate data point for reporting, given that over 90% of patients in both the PMCT + CDT and PMCT + CDT + stent groups underwent ultrasonographic follow-up at 24 months. Venous vessel and stented venous vessel patency were evaluated using ultrasound and computed tomography (CT). Various parameters, including lower-extremity venous caliber, patency, compressibility, thrombus extent, and valvular reflux, were assessed. Additionally, the patency of the iliofemoral segment was routinely evaluated via CT venography examination after 6 months for the PMCT + CDT + stent group. Ultrasonography was employed to examine phasic flow at the level of the common femoral vein for both the PMCT + CDT and PMCT + CDT + stent groups.

For the purposes of this study, post-thrombotic syndrome (PTS) diagnosis relied on the presence of a minimum of five symptoms or clinical signs as outlined in the Villalta score descriptors. The venous reflux examination protocol assessed valve reflux at the common femoral, superficial femoral, and popliteal levels. The pathological valvular reflux was determined if the valve closure time exceeded 0.5 sec in superficial veins and if the valve closure time exceeded 1.0 sec in femoral and popliteal veins.

Patient outcome was assessed with Villalta scores calculated at six months, one year, and two years. Severity was defined as a Villalta score of 0–4 (no PTS), 5–9 (mild PTS), 10–14 (moderate PTS), and 15 or the presence of ulceration (severe PTS).

A retrospective evaluation of electronic patient records was conducted for the analysis of lytic dose and duration and for the incidence of complications. Safety outcomes measured included minor bleeding (epistaxis, gum bleeding, menorrhagia, and hematoma) and major bleeding (retroperitoneal and hemorrhagic stroke); documented hemoglobinuria; clinical diagnosis of acute kidney injury; allergic reaction; cardiac event; bacteremia; limb loss; and death.

Follow-up duplex ultrasonography was conducted at multiple time points: one day, two weeks, six weeks, three months, six months, twelve months, eighteen months, and, finally, twenty-four months post-treatment. Re-interventions were carried out in cases where there was a reduction in vessel diameter of more than 50% or complete occlusion accompanied by the recurrence of symptoms. Primary patency referred to continuous vessel patency (less than 100% stenosis) without requiring re-intervention. Primary-assisted patency indicated sustained vessel patency with re-intervention but no complete occlusion due to vessel thrombosis. Secondary patency was defined as patency restored following re-intervention due to vessel thrombosis occlusion.

Secondary outcomes assessed included both major and minor bleeding complications occurring during hospitalization. Major bleeding events encompassed intracranial and gastrointestinal bleeding, as well as the need for transfusion of 2 units or more of packed red blood cells. Minor bleeding complications involved sheath hematoma and ecchymosis. Additionally, other secondary outcomes examined were in-hospital mortality and the occurrence of renal failure.

### 2.4. Statistical Analysis

The patients’ data have been analyzed as percentages for categorical data and as median range or interquartile range (IQR) for non-parametric continuous variables. Continuous non-parametric variables were compared using Mann–Whitney U or Kruskal–Wallis tests. Chi-square test with Yates’ correction or Fisher’s exact test was employed for categorical data. A *p*-value of <0.05 was deemed statistically significant, and no adjustment was made for multiple significance testing. Survival analysis of vessel patency was performed using the Kaplan–Meier method and log-rank Mantel–Cox test.

## 3. Results

A total of 63 patients underwent PMCT + CDT (comprising 38 (60.4%) women), with a mean age of 58.42 ± 16.22 years. Additionally, 49 patients received treatments involving PMCT + CDT + iliac vein stenting (among whom 29 (59.2%) were women), with a mean age of 61 ± 12.9 years. The details of the presenting symptoms and physical examination findings can be found in Table 1. All patients were initially diagnosed with acute DVT. Thirty-five patients (%31.2) had only iliac vein thrombosis, while seventy-seven patients (%68.8) had iliofemoral vein thrombosis. The observed symptoms encompassed edema (%100), pain (%86.6), venous claudication (%76.7), hyperpigmentation (%6.2), and ulceration (%3.5) across all patients. The median time to clinical symptom appearance was 6 days (IQR 2–11). The differences in patient features, risk factors, and thrombus extension between the two groups were statistically insignificant (Table 1).

### 3.1. Patient Outcomes

**Figure 1:**
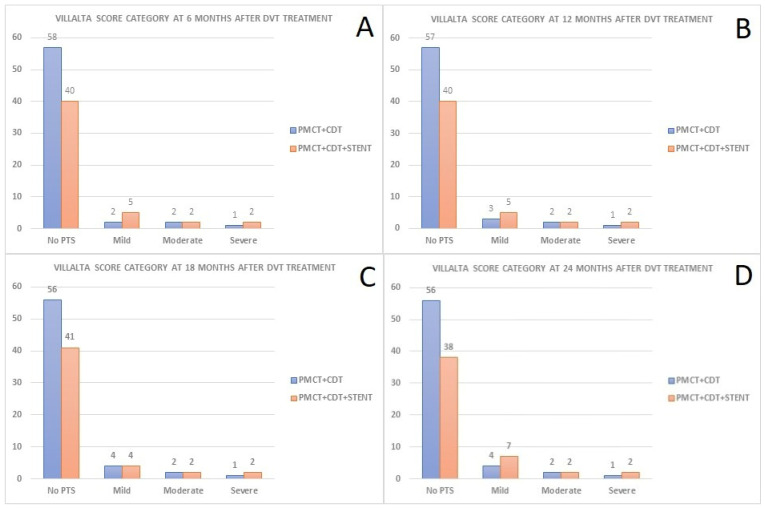
Number of patients with each Villalta score category at (**A**) 6 months, (**B**) 12 months, (**C**) 18 months, and (**D**) 24 months after DVT treatment. PMCT: pharmacomechanical catheter-directed thrombolysis; CDT: catheter-directed thrombolysis; PTS: post-thrombotic syndrome.

The primary outcome, as evaluated by the 6-month, 12-month, 18-month, and 24-month Villalta scores, showed no statistically significant difference between the two groups (Table 2, Figure 1). In the PMCT + CDT group, three patients experienced moderate or severe PTS, while four patients in the PMCT + CDT + stent group had moderate or severe PTS.

### 3.2. Procedural Outcomes

The treatment time did not significantly differ between groups, with similar usage of treatment modalities (32.87 ± 11.98 h vs. 35.39 ± 13.54 h; *p* = 0.267). The dosage of the thrombolytic regimen was also comparable, with values of 31.76 ± 12.57 mg vs. 36.81 ± 13.74 mg (*p* = 0.432). Additionally, the lytic duration time for both groups was similar (18.43 ± 5.73 h vs. 20.03 ± 7.52 h; *p* = 0.861). Further procedural details can be found in Table 3.

### 3.3. Safety Outcomes

This study was not sufficiently powered to detect differences in major and minor bleeding events. However, both treatment groups receiving thrombolysis therapy experienced bleeding complications. In the PMCT + CDT group, there were three cases of gastrointestinal bleeding, compared to two cases in the PMCT + CDT + stent group (*p* = 0.900). Additionally, one intracranial hemorrhage occurred in the PMCT + CDT group and two in the PMCT + CDT + stent group. Unfortunately, one patient in the PMCT + CDT group died due to intracranial bleeding, while the other two patients with intracranial hemorrhage complications were discharged following the procedure. Notably, no cases of pulmonary embolism (PE), in-hospital mortality, or other fatal complications were observed in either group during treatment. In the PMCT + CDT alone group, three cases (4.7%) of minor-access puncture-site hematoma were observed, with two of them also experiencing catheter-related infection. Similarly, in the PMCT + CDT + stent group, there were also three cases (6.1%) of minor-access puncture-site hematoma. Despite a higher incidence of hemorrhage in the PMCT + CDT alone group, blood transfusion was more common in the PMCT + CDT + stent group (Table 4).

There were no significant differences observed in the length of stay (*p* = 0.214). Additionally, a few patients experienced acute recurrence within one month. Specifically, three patients in the PMCT + CDT alone group and one patient in the PMCT + CDT + stent group had acute recurrence. Although symptom resolution appeared to be at a higher rate in the PMCT + CDT + stent group compared to the PMCT + CDT alone group, this difference was not statistically significant.

Renal function damage was observed in one patient in the PMCT + CDT group on the second day of the intervention, as evidenced by a 50% increase in creatinine levels; however, hemodialysis was not required, fortunately. Additionally, there was an increase in hemoglobinuria following the interventions in both treatment groups, with 39 out of 63 patients (39/63) in the PMCT + CDT group and 26 out of 49 patients (26/49) in the PMCT + CDT + stent group experiencing this phenomenon. However, there was no statistically significant difference observed between the two groups (*p* = 0.724). The duration of hematuria was recorded as 7.19 ± 3.27 h in the PMCT + CDT group compared to 6.92 ± 4.13 h in the PMCT + CDT + stent group (*p* = 0.687) (Table 4).

### 3.4. Vessel Patency

**Figure 2:**
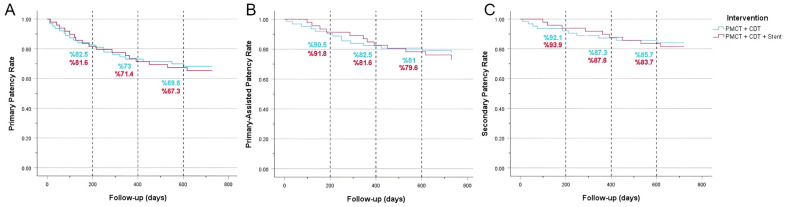
Cumulative Kaplan–Meier estimates for primary (**A**), primary-assisted (**B**), and secondary (**C**) patencies of pharmacomechanical catheter-directed thrombolysis and catheter-directed thrombolysis (PMCT + CDT) vs. pharmacomechanical catheter-directed thrombolysis, catheter-directed thrombolysis, and stenting (PMCT + CDT + stent) for the treatment of symptomatic acute iliofemoral deep venous thrombosis (DVT). There was no significant difference between intervention methods for primary patency (*p* = 0.74), primary-assisted patency (*p* = 0.59), or secondary patency (*p* = 0.72).

At 6 months, there was no difference in the observed cumulative patency rates, with rates standing at 82.5% for PMCT + CDT and 81.6% for PMCT + CDT + stent. Survival analyses for primary, primary-assisted, and secondary patency yielded comparable results between PMCT + CDT and PMCT + CDT + stent, with *p*-values of 0.74, 0.59, and 0.72, respectively (Figure 2).

In this study, we also evaluated the long-term outcomes of patients. The two-year patency rate was high in both groups (85.7% for PMCT + CDT vs. 83.7% for PMCT + CDT + stent). At 24 months post-intervention, the incidence of post-thrombotic syndrome (PTS) was 11.1% in the PMCT + CDT group and 22% in the PMCT + CDT + stent group (*p* = 0.381) (see Figure 2). Additionally, the overall mortality was 3.1% in the PMCT + CDT group and 6.1% in the PMCT + CDT + stent group.

## 4. Discussion

In this study, percutaneous interventions involving both PMCT + CDT and PMCT + CDT + stent demonstrated favorable patient outcomes. The overall incidence of post-thrombotic syndrome (PTS) at one year was only 13.3%, with rates of 9.5% for PMCT + CDT and 18.4% for PMCT + CDT + stent. The average Villalta score was 2.65 for the PMCT + CDT group and 3.51 for the PMCT + CDT + stent group, although this difference was not statistically significant (*p* = 0.361). Additionally, both treatment modalities showed similar vessel patency rates, with no significant differences observed in primary, primary-assisted, or secondary patency at the two-year follow-up. The average Villalta score was 3.21 for the PMCT + CDT group and 4.02 for the PMCT + CDT + stent group, with no significant difference found (*p* = 0.318).

The observed rates of PTS in our study were notably lower compared to those reported in previous trials [2,3,4,5,6,7]. For instance, in the Catheter-Directed Venous Thrombolysis in Acute Iliofemoral Vein Thrombosis (CaVent) trial, the incidence was 41.1% for CDT vs. 55.6% for no CDT, and in the ATTRACT trial, it was 49% for PCDT vs. 51% for no PCDT.

Additionally, in our study, only 2.9% of cases were classified as moderate to severe in terms of PTS, which was significantly lower than the rates observed in the ATTRACT trial (18% for PCDT vs. 28% for no PCDT). This favorable outcome could be attributed to a clear treatment protocol, including timely intervention, the use of combined thrombectomy and thrombolysis modalities, appropriate stent treatment tailored to each patient, and frequent post-procedure monitoring, thereby ensuring the maintenance of an open vein at the two-year follow-up [8].

Utilizing mechanical thrombectomy and catheter-directed thrombolytic therapy in conjunction enables effective clot dissolution. The CDT treatment modality plays a critical role in achieving successful intervention, especially when the thrombus is extensive or resistant to PMCT. In cases of acute iliofemoral DVT, CDT + PMCT therapy followed by iliac vein stenting is an important treatment option for select patients. However, previous studies have often focused on comparing different treatment modalities without considering the importance of thrombus removal prior to stenting [9,10,11,12,13,14,15,16,17,18,19,20]. Thrombus removal should be adequately performed before stenting. During combined treatment modalities such as PMCT + CDT, the goal is to reduce residual lesions or thrombus burden. CDT for iliofemoral DVT is increasingly performed, particularly in symptomatic patients with low bleeding risk [15,16,17]. Mewissen et al. reported that 1-year venous patency was significantly better in limbs treated with iliac stents (74%) than in limbs without stent placement (53%; *p* < 0.001) [18]. Complete removal of the acute thrombus is crucial, as incomplete thrombolysis or residual diseased vein segments may negatively affect long-term stent patency. In this study, unlike previous research, effective long-term outcomes were achieved with PMCT + CDT treatment, regardless of stent placement. The two-year patency rate was high in both groups (85.7% for PMCT + CDT vs. 83.7% for PMCT + CDT + stent) (Figure 2) [20,21,22,23].

The extension of stents into the infrainguinal region has previously been associated with a potential decrease in stent patency, despite the fact that overall patency rates remain relatively high. In a recent series focusing on deep vein thrombosis (DVT) stenting, there was no observed discrepancy in primary patency at the 12-month mark between individuals who had stenting limited to the suprainguinal area and those necessitating extension into the infrainguinal region (68% vs. 65%; *p* = 0.7) [19].

Liberal stenting of residual lesions is important for better outcomes. Razavi et al., in their 2015 meta-analysis of iliac vein stenting, described that the primary patency of stents after PMCT ranges between 79% and 100% at 1 year and between 60% and 90% at 5 years [20,21,22,23,24]. Stent thrombosis is a really significant complication because of the potential for the development of PTS. Park et al. found that limbs with more than 6 cm of stented vessels were 13 times more likely to experience rethrombosis after undergoing CDT and stenting compared to those with shorter treated segments [25].

A number of retrospective trials have shown that iliocaval stenting for the treatment of acute DVT is safe and effective. The rationale for stenting is to address the underlying cause of the DVT, typically an iliac vein compression, known as a May–Thurner lesion, or to cover residual acute and chronic thrombotic–collagenous material that causes significant stenosis (more than 70% diameter stenosis). This assumption is primarily derived from studies on chronic obstructive disease. Raju et al. described 504 patients (528 limbs) who underwent iliac vein stenting [26]. Pain and swelling improved by 78%, the ulcer healing rate was 54%, and freedom from ulcer recurrence was 88% at 5 years. Major adverse events have not been seen often. Jayaraj et al. [27] evaluated 65 patients (69 limbs) with acute symptoms of iliofemoral deep vein thrombosis. In their study, 53% of patents did not require stenting over the duration of the follow-up, with a native vein patency of 72%. Bleeding complications requiring transfusion occurred in this study.

Although no trial offers a direct comparison between stenting and no stenting after early thrombus removal, expert opinion and indirect evidence support this practice. It aligns with the general principle of ensuring good inflow and outflow in any deep venous intervention to restore iliac vein patency [28]. Several causes may affect patency, including incomplete thrombus clearance due to partial aging of the thrombosis and stent placement too low to fully cover the iliac venous stenosis lesion segment [29].

The patency of the iliofemoral venous outflow post-intervention has been identified as an independent risk factor for PTS and thrombus recurrence. Ming et al. reported that the incidence of PTS at 12 months of follow-up was 8.0% in patients with stent implantation, compared to 37.1% in those without [29]. Additionally, they emphasized that the strategy of direct or staged iliac vein stent implantation can significantly influence the long-term patency rate of the vein. Liu et al. demonstrated that iliac vein stent implantation effectively reduces the length of hospital stay and enhances the long-term patency rate [22]. Furthermore, studies have demonstrated long-term improvement in post-thrombotic syndrome (PTS) rates following stenting in CDT [27]. It is thought that more than 50% of cases of acute iliofemoral DVT should undergo additional stenting alongside CDT [5,6,9]. The stenting rate in the Catheter-Directed Venous Thrombolysis in Acute Iliofemoral Vein Thrombosis (CaVenT) trial was only 18%, and in the Acute Venous Thrombosis: Thrombus Removal with Adjunctive Catheter-Directed Thrombolysis (ATTRACT) trial, it was only 39% of iliofemoral DVT patients [5,6]. The limited utilization of IVUS in evaluating residual outflow vein stenosis likely contributed to the low rate of stenting observed in these trials [30,31]. Therefore, these trials have been criticized for failing to show the superiority of catheter interventions.

In Tang et al.’s analysis, adjunctive stenting with CDT was linked with higher charges but did not prolong the length of stay. They claimed that if the complications from DVT can be prevented with adjunctive stenting, the overall cost of this condition may be reduced [32].

Several limitations are notable in our study. First, our study is limited by its single-center retrospective case–control design and small sample size. Second, the results may be susceptible to consecutive-case selection bias, although this is not expected to influence the results. Third, the lack of objective tools available (such as IVUS, not available in our country) during the procedure may potentially reduce the clearance of the outcomes. Finally, conducting a multi-center, randomized controlled trial will be necessary to thoroughly test and compare the safety and efficacy of CDT and PMCT for treating iliofemoral DVT.

## 5. Conclusions

PMCT + CDT and PMCT + CDT + stent showed comparable long-term outcomes. The overall low incidence of PTS and the notable decrease in the number of patients experiencing moderate-to-severe PTS imply that modern combined techniques of clot removal and inline venous flow reconstruction may offer benefits to patients with acute iliofemoral DVT.

## Figures and Tables

**Table 1:** Patients’ characteristics and clinical presentation.

	PMT + CDT (*n* = 63)	PMT + CDT + Stent (*n* = 49)	*p*
Age (years, mean ± SD)	58.42 ± 16.22	61.45 ± 12.9	0.546
Male (*n*; %)	25(39.8)	20(40.8)	0.234
Female (*n*; %)	38(60.2)	29(59.2)	0.467
Risk Factors (*n*; %)			
Previous DVT	6(9.5)	6(12.2)	0.452
Thrombophilia	21(33.3)	19(38.7)	0.322
Cancer	15(23.8)	7(14.3)	0.631
Recent surgery and trauma BMI > 30	21(33.3) 19(30.1)	17(34.7)	0.345
Affected limb (*n*; %)			
Right	14(22.2)	16(32.6)	0.123
Left	49(77.8)	33(67.4)	0.268
Thrombosis location (*n*; %)			
Iliofemoral	39(61.9)	38(77.5)	0.167
Iliac	24(38.1)	11(22.4)	0.098
Clinical presentation (*n*; %)			
Edema	63(100)	49(100)	0.479
Pain	53(84.1)	44(89.8)	0.432
Venous claudication	47(74.6)	39(79.6)	0.433
Hyperpigmentation	4(6.3)	3(6.1)	0.343
Ulceration	3(4.7)	1(2)	0.34

Data are presented as *n* (%) or mean ± standard deviation. DVT: deep vein thrombosis.

**Table 2:** Patients’ Villalta scores.

	PMCT + CDT (*n* = 63)	PMCT + CDT + Stent (*n* = 49)	*p*-Value
Villalta Score (6 Months)			
No PTS	58	40	
Mild	2	5	
Moderate	2	2	
Severe	1	2	
Average Score	2.65	3.38	*p* = 0.283
Villalta Score (12 Months)			
No PTS	57	40	
Mild	3	5	
Moderate	2	2	
Severe	1	2	
Average Score	2.89	3.51	*p* = 0.361
Villalta Score (18 Months)			
No PTS	56	41	
Mild	4	4	
Moderate	2	2	
Severe	1	2	
Average Score	3.01	3.49	*p* = 0.196
Villalta Score (24 Months)			
No PTS	56	38	
Mild	4	7	
Moderate	2	2	
Severe	1	2	
Average Score	3.21	4.02	*p* = 0.318

Data are presented as *n* (%) or mean ± standard deviation. PTS: post-thrombotic syndrome.

**Table 3:** Perioperative outcomes categorized by treatment group.

	PMT + CDT (*n* = 63)	PMT + CDT + Stent (*n* = 49)	*p*
Treatment time, hours	32.87 ± 11.98	35.39 ± 13.54	0.267
Dosage of rTPA, mg	31.76 ± 12.57	36.81 ± 13.74	0.432
Lysis duration time, hours	18.43 ± 5.73	20.03 ± 7.52	0.861
D-dimer on admission, μg/L	12,479.38 ± 5391.42	11,347 ± 6793.71	0.981
D-dimer at discharge, μg/L	524.34 ± 865.35	478.81 ± 621.58	0.345
Length of stay, days	6.34 ± 2.12	6.78 ± 3.45	0.214
Acute recurrence (within 1 month)	3(4.7)	1(2.1)	0.850

**Table 4:** Safety outcomes and incidence of procedural complications.

	PMT + CDT (*n* = 63)	PMT + CDT + Stent (*n* = 49)	*p*
Death	2(3.2)	3(6.1)	0.739
***Fatal Bleeding***			
Intracranial hemorrhage	1(1.6)	0	
***Major Bleeding***			
GI bleeding	3(4.7)	2(4.1)	0.909
Blood transfusion	4(6.3)	6(12.2)	0.567
Intracranial hemorrhage	0	2	
***Minor Bleeding***			
Hematoma	3(4.8)	3(4.8)	0.916
Hemorrhage	7(11.1)	5(10.2)	0.875
Renal function damage	1(1.6)	0	0.939
Infection	2(3.2)	1((2)	0.922
Hemoglobinuria	39(62)	26(53.1)	0.724
Duration of hematuria (hours)	7.19 ± 3.27	6.92 ± 4.13	0.687
PTS at 2 years	7(11.1)	11(22.4)	0.381

## Data Availability

Data are contained within the article.
